# Improved Method of Packing Moulds

**Published:** 1874-09

**Authors:** Geo. S. Fouke

**Affiliations:** Westminster, Md.


### ARTICLE III.

### *Improved Method of Packing Moulds.*

BY GEO. S. FOUKE, WESTMINSTER, MD.

The prominence of the alveolar ridge and the impinging of the teeth and gums upon the cast, very often complicate the packing of the mould to a degree that interferes materially with the success and integrity of the work. This difficulty is entirely overcome, and the closing of the flask made easy and sure by packing or introducing the material of which the plate is formed directly against the male cast, and by retaining the teeth and the cast in their true relative position to each other.

THE PROCESS.—Place the case in the bottom of the flask in the usual way, and cover up the rim with plaster to the top of the gums, trimming as usual. Oil the plaster around the outside of teeth and across the back of the flask, place the ring on the bottom of the flask and proceed to fill with plaster in the following manner:

Pour the plaster carefully around the teeth until the space between the teeth and ring is filled up, and finish this part of the mould by covering up the tops of the teeth out flush with the lingual surfaces, and by building up the plaster from the edge of the base plate in the rear up all round to the edge of the flask. Trim the plaster from the edge of the base-plate and border of the lingual surfaces of the teeth with a sufficient bevel and roundness to allow of an easy separation of the top part, (to be moulded afterwards,) from the ring part of the mould. Having filled the ring part of the mould as directed above, and trimmed the plaster properly, oil the entire surfaces of the base-plate and plaster, fill up the mould with plaster, and adjust the top plate of the flask to its place.

Separating the top-piece and bottom from the ring of the flask, and removing the base-plate, proceed to make very small neatly cut gates; as the quantity of material required to fill the mould is so accurately and definitely ascertained that scarcely any vent is necessary for any excess of the rubber used. For cutting the gates a sharp > curve pointed scraper is best adapted to the purpose. When ready to pack remove the bottom piece first and fill with rubber the *rim* portion of the mould, return it to its place, and with the screw clamp press it till adjusted to its place against the ring. Next remove the top piece and proceed to fill all the spaces between the cast and the teeth, till the pins of the teeth are well covered and the mould is packed out flush with the lingual sides of the teeth. A piece of rubber large enough to cover the face of the cast, and a few extra pieces of rubber used where it is indicated to be required, and the top-piece of the flask is pressed down against the rubber, using for the purpose a large screw clamp. The top had best be removed now, and any deficiency of rubber noticed can be supplied, when the final closing of the flask is made, the screws applied and the operation completed.

The dentist will readily perceive the great practical advantages of this process of packing the mould, in the exact and certain progress of the work, and in the avoidance of injury to the teeth or any derangement of their articulation.

It may be added that in cases where the teeth rest hard on the ridge, (especially in partial cases,) and no rim exists, the ring and bottom of the flask may be confined together by setting the case in plaster in the bottom of the flask, placing on the ring and covering the teeth at once. In this way there will be but one point of separation, namely, the top-piece from the other part of the mould.

It is believed the extreme simplicity of this method of packing the mould, and the entire accuracy resulting from it, will enable the dentist to continue the use of the flasks and vulcanizers as now constructed, and avoid the expense and trouble of the complicated devices recommended for this purpose.

---

### SELECTED ARTICLES.

